# Estimating LoD-s Based on the Ionization Efficiency Values for the Reporting and Harmonization of Amenable Chemical Space in Nontargeted Screening LC/ESI/HRMS

**DOI:** 10.1021/acs.analchem.4c01002

**Published:** 2024-07-03

**Authors:** Amina Souihi, Anneli Kruve

**Affiliations:** † Department of Environmental and Materials Chemistry, Stockholm University, Svante Arrhenius väg 16, 106 91 Stockholm, Sweden; ‡ Department of Environmental Science, Stockholm University, Svante Arrhenius väg 8, 106 91 Stockholm, Sweden

## Abstract

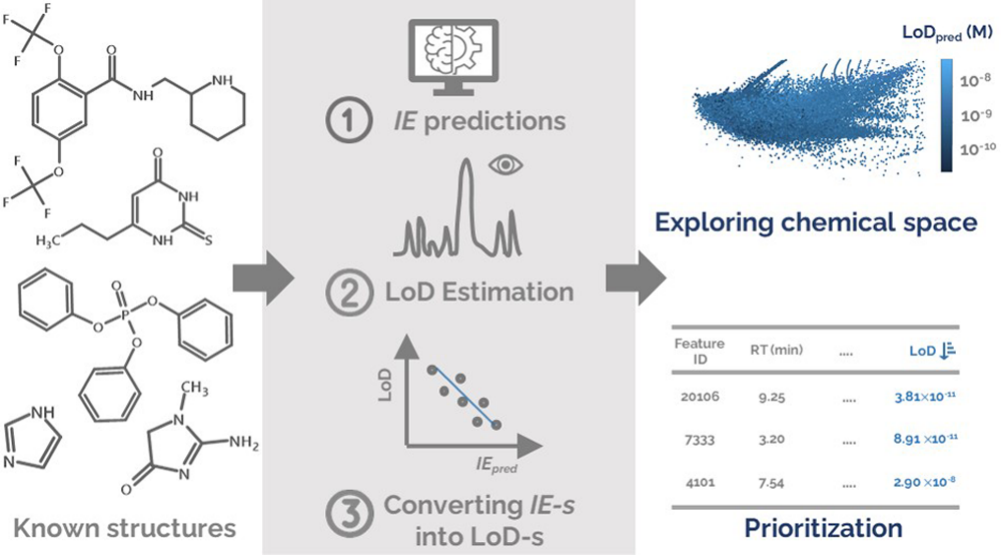

Nontargeted LC/ESI/HRMS aims to detect and identify organic compounds present in the environment without prior knowledge; however, in practice no LC/ESI/HRMS method is capable of detecting all chemicals, and the scope depends on the instrumental conditions. Different experimental conditions, instruments, and methods used for sample preparation and nontargeted LC/ESI/HRMS as well as different workflows for data processing may lead to challenges in communicating the results and sharing data between laboratories as well as reduced reproducibility. One of the reasons is that only a fraction of method performance characteristics can be determined for a nontargeted analysis method due to the lack of prior information and analytical standards of the chemicals present in the sample. The limit of detection (LoD) is one of the most important performance characteristics in target analysis and directly describes the detectability of a chemical. Recently, the identification and quantification in nontargeted LC/ESI/HRMS (e.g., via predicting ionization efficiency, risk scores, and retention times) have significantly improved due to employing machine learning. In this work, we hypothesize that the predicted ionization efficiency could be used to estimate LoD and thereby enable evaluating the suitability of the LC/ESI/HRMS nontargeted method for the detection of suspected chemicals even if analytical standards are lacking. For this, 221 representative compounds were selected from the NORMAN SusDat list (S0), and LoD values were determined by using 4 complementary approaches. The LoD values were correlated to ionization efficiency values predicted with previously trained random forest regression. A robust regression was then used to estimate LoD values of unknown features detected in the nontargeted screening of wastewater samples. These estimated LoD values were used for prioritization of the unknown features. Furthermore, we present LoD values for the NORMAN SusDat list with a reversed-phase C_18_ LC method.

## Introduction

Targeted LC/ESI/HRMS is commonly used for monitoring contaminants in environmental samples (e.g., drinking, surface, and wastewater). This method enables the detection and quantification of a limited number of chemicals and requires the use of analytical standards in method development, identity confirmation, and quantification. Therefore, nontargeted screening (NTS) using LC/ESI/HRMS is increasingly used as an advantageous alternative to detect hundreds or even thousands of chemical features.^1^ The most relevant of the detected features can be prioritized for identification and quantification with analytical standards depending on the research question. Nontargeted LC/ESI/HRMS screening aims to reveal all chemicals present in the sample; however, there is no method capable of that.^2^ Depending on the chromatographic conditions, ionization source, acquisition methods, and sensitivity of the instrument, only a subset of the chemicals in the sample can be detected.^2^ Most laboratories use nonstandardized nontargeted LC/ESI/HRMS methods utilizing different chromatographic conditions and acquisition modes;^2^ therefore, the scope of LC/ESI/HRMS methods might be significantly different and the results from NTS might be hard to reproduce or compare.^3^ This furthermore results in a low comparability of NTS results between different laboratories.^4^ For example, 2350 compounds were tentatively identified in an interlaboratory comparison led by Rostkowski et al.;^5^ however, less than 40% of these compounds were reported by more than one laboratory. The reasons for the low number of commonly reported compounds and lack of reproducibility are hard to pinpoint due to the absence of performance characteristics.^3^ Performance characteristics, such as accuracy, sensitivity, precision, and selectivity, are central to the assessment of the targeted analytical methods; however, analytical standards of the chemicals are required to evaluate these characteristics^3,6^ and thus these characteristics remain unknown for NTS.

The NTS Study Reporting Tool (SRT) was recently developed by the group of Benchmarking and Publications for Nontargeted Analysis (BP4NTA)^7^ to evaluate, standardize, and harmonize the quality of reporting as well as improve the study design.^8^ Nevertheless, it has been shown that more improvements are needed regarding quality assurance and quality control information.^8^ Black et al.^9^ proposed ChemSpace, a tool for exploring the chemical space in NTS, involving multiple filtering steps that reduce the suspect lists (e.g., databases) into a list of possibly detectable compounds. In this case, the filtering-based approach might be too harsh since the compound detectability is not an on–off switch. Rather, the detectability is a combination of method sensitivity and analyte concentration.

The limit of detection (LoD) is one of the most important parameters of the method performance that affects the interpretation of data. In different validation guides for target analysis, the limit of detection has been suggested to be determined using different approaches.^10^ One of the most commonly used approaches is based on evaluating the signal of the analyte at different concentrations with either (1) the signal-to-noise ratio or (2) the cut-off approach. The first approach uses a signal-to-noise ratio (S/N) limit, usually 3, to consider whether the analyte is detected. The limitation of this approach is that false positives (analyte is wrongly detected) and false negatives (analyte is wrongly not detected) are not explicitly considered. Therefore, the cut-off approach is considered to be more robust and reliable where a set of concentrations (usually at least 10 concentrations) are analyzed, and the lowest detected concentration is considered to be the LoD. In this case, the possibility of false positives is considered, and no prior assumptions are made. Another approach for LoD determination is based on considering the LoD equivalent to the decision limit (CC_α_) which considers only false positive results. In this case, the LoD is calculated using the standard deviation of analyte concentration from *t* replicate measurements (*t* is usually between 1 and 5).^10^ Finally, the LoD could be estimated using the slope, standard deviation of residuals and the intercept from a calibration graph in the LoD range. This approach has been shown to yield a conservative estimate of the LoD and can therefore be used if a robust LoD estimate is not required.^10^ In spite of the variety of methods existing for LoD determination in target screening, approaches for evaluating the LoD in NTS are yet to be suggested.

The LoD can vary significantly between days^11,12^ due to the cleanliness of the LC/ESI/HRMS system and small differences in the mobile phase pH. In some cases, the difference in LoD, up to 10×, has been reported between days.^10,13^ Still, within the same day or sequence, the LoD depends on the sensitivity of the method and the instrument toward the analyte and background noise at a given *m*/*z*, meaning that the LoD increases with decreasing sensitivity. The sensitivity can be represented as the slope of the calibration curve that is largely impacted by the ionization efficiency of the chemicals in ESI/HRMS.^14^ Recently, different machine learning models have been proposed to predict the ionization efficiency from the molecular descriptors and mobile phase composition.^15−20^ This opens an avenue for studying the LoD for chemicals that lack analytical standards in NTS based on their predicted ionization efficiency values.

In this study, we evaluate the possibility of estimating the LoD of chemicals detected with LC/ESI/HRMS based on the predicted ionization efficiency values. For this purpose, 221 representative compounds were selected from the NORMAN SusDat list (S0)^21^ using a principal component analysis, and the LoD-s were determined for these compounds using four approaches: (1) cut-off, (2) extrapolating the S/N of the lowest concentration detected, (3) residuals of the four lowest concentrations in the calibration curve, and (4) using the standard deviation of the peak area of the lowest detected concentration in the calibration curve. Furthermore, we correlate the LoD-s with the experimental calibration graph slopes and predicted ionization efficiency values. Finally, we fit and use regression models to estimate the LoD-s of unknown features detected in real wastewater samples. The LoD-s can be used further for feature prioritization or comparison of NTS methods across laboratories.

## Experimental Section

### Selection of Representative Compounds

NORMAN SusDat list (S0), version 0.3.2 from Feb 23, 2021,^21^ contains more than 100,000 compounds that are relevant in the context of screening environmental samples (e.g., surface and wastewater samples). Initially, compounds missing a carbon and nitrogen/oxygen or having a *m*/*z* below 100 Da were filtered out from the S0 list because they are unlikely to be detected with LC/ESI/HRMS. For all 70,259 remaining compounds in S0, 1217 PaDEL descriptors^20^ and log*P* values (*rcdk* package^21^) were calculated. Principal component analysis (PCA) was conducted on the calculated PaDEL descriptors and log*P* values. The first two principal components, representing 14% of the variability, were plotted to select a representative set of 221 compounds for this study (Figure S1 and Table S1). The selection of compounds aimed for maximum spread over the whole chemical space (S0) according to the first and second principal components; however, structures, masses, and commercial availability of the selected compounds were checked manually alongside expert knowledge of detectability in LC/ESI/HRMS and functional groups enabling ionization in ESI.

In addition, the NORMAN SusDat list (S0) was used as a representation of chemical space relevant to the wastewater samples. The S0 list contains the probabilities of amenability in positive ESI predicted by Alygizakis et al.^22^ These probabilities were compared with the LoD values predicted in this work. Another list of 2657 compounds compiled by Hulleman et al.^23^ was used to evaluate the detectability of the nontargeted LC/ESI/HRMS method by estimating the LoD-s. The list contains compounds identified at level 1 or 2 on a scale defined by Schymanski et al.^24^ in different studies published between 2017 and 2023 focusing on NTS with LC/HRMS.

### Solvents

HPLC-grade water, acetonitrile, and methanol were purchased from Sigma-Aldrich. LC-MS-grade formic acid was purchased from Merck and was used as an additive to prepare the aqueous phase: 0.1% formic acid (pH = 2.7) for ESI+.

### Standard Solutions and Wastewater Samples

All 221 compounds (Table S1) were prepared in individual stock solutions and combined into a spiking solution with an approximate concentration of 1000 μg/L [1.47 × 10^–5^–6.91 × 10^–7^ M]. Sixteen dilutions were prepared at the following approximate concentrations: 500, 100, 50, 10, 5, 1, 0.5, 0.1, 0.05, 0.01, 0.005, 0.001, 0.0005, 0.0001, 0.00005, and 0.00001 μg/L [7.60 × 10^–15^–7.83 × 10^–6^ M]. All solutions contained 20% methanol, and the exact concentrations obtained from weighing were used in the following LoD calculations.

Influent and effluent wastewater samples were provided from a wastewater treatment plant in the Stockholm region. A pooled sample was prepared by mixing the influent and effluent samples in a 50:50 ratio. Methanol (20%) was added to the samples, and 16 samples spiked at the same concentration levels as the standard solutions were prepared. The standard solutions were analyzed in randomized order, followed directly by another sequence of wastewater samples also run in a randomized order.

The comparison of the automatic integration workflow with manual integration was performed on a smaller batch of standard solutions in five different mixtures. The concentration levels were 1000, 500, 100, 50, 10, 5, 1, 0.1, 0.05, and 0.01 μg/L [8.96 × 10^–6^–1.33 × 10^–9^ M].

### Instrumental Analysis

Q Exactive Orbitrap HRMS (Thermo Fischer Scientific, USA) was used together with a Dionex UltiMate 3000 UPLC (Thermo Fischer Scientific, USA) system for the analysis of the standard solutions and wastewater samples. For the first batch of standard solutions and wastewater samples, an ACQUITY UPLC HSS T3 VanGuard precolumn (100 Å, 1.8 μm, 2.1 mm × 5 mm, Waters, Ireland) was used and corrected to an Acquity ULPC HSS T3 column (100 Å, 1.8 μm, 2.1 mm × 100 mm, Waters, Ireland). The method was 13 min long and used a 0.1% formic acid water phase (A) and acetonitrile (B). The percentage of B started at 5%, increased gradually to 95% over 10 min, stayed constant for 2 min, and finally dropped to 5% in 0.1 min. Standard solutions and wastewater samples (100 μL each) were injected at a flow rate of 0.4 mL/min. Positive ESI mode was used in the range of 100 up to 1500 Da with a nominal mass resolution of 120 000 and an MS^2^ nominal resolution of 15 000. Data-dependent MS^2^ acquisition was used with a *m*/*z* inclusion list of 221 selected compounds, together with top 5 intensity-based approach as well as dynamic exclusion list for 5 s. The probe heater and capillary temperature were set to 350 and 320 °C, respectively. The spray voltage was set to 3500 V.

The standard solutions for the comparison of automatic and manual integration were analyzed with a Kinetex 2.6 μm PS C18 100 Å (150 × 4.6 mm^2^). Positive mode ionization with the following settings was used: capillary voltage of 3.2 kV, cone voltage of 40 V, source temperature of 150 °C, desolvation temperature of 600 °C, cone gas flow of 60 L/h, and desolvation gas flow of 1000 L/h. Gradient elution started with a 95:5 0.1% formic acid water phase and acetonitrile, followed by a linear increase to 100% acetonitrile over 20 min that was finally kept at 100% for 5 min. The system was equilibrated with 95:5 0.1% formic acid water phase and acetonitrile for 5 min between injections.

### Data Processing

For the automatic integration workflow, the peak picking and extraction of MS^2^ spectra of protonated species were performed with open-source MS-DIAL^25^ version 4.9. Data collection was performed using mass tolerances of 0.01 and 0.025 Da in MS^1^ and MS^2^, respectively. Retention times were accepted between 0 and 13 min. The parent mass range in MS^1^ was 100 to 1500 Da, and the fragment mass range in MS^2^ was from 0 to 1500 Da. Chlorinated and brominated molecular formulas were considered, and a maximum charge of 2 was allowed. For the peak detection, a minimum peak height of 10 000 and a mass slide width of 0.05 Da were used. The sigma window value was set to 1, and the MS^2^ abundance cutoff was set to 0 amplitude. MS2Dec was excluded after the precursor ion, and the isotopic ions were kept until 5 Da. The retention time tolerance in alignment was set to 0.1 min, and the MS^1^ tolerance to 0.015 Da. The features with intensity 5× higher than the blank intensity and detected at least in two of the triplicates were considered for further analysis.

For the manual integration workflow, a Thermo Xcalibur Processing Setup Quan identification and browser (Thermo Fisher Scientific, Waltham, MA, USA) were used. The peaks of the protonated ions were checked and integrated manually with a mass tolerance of 10 ppm.

### Data Analysis

R version 4.3 and RStudio version 2023.03.0+386 were used for the data handling and visualization. The response factor refers to the slope of the calibration curve of each compound. It was calculated using the *coef()* function to extract the coefficients of the linear model. An in-house R-script code was used to perform the estimation of LoD using a while loop. In the linear range determination, all of the peak areas and concentrations were initially used to build the calibration curve. In every iteration, the lowest concentration was excluded from the calibration curve, and the absolute relative residuals were recalculated and compared to 5%. The loop ended when all values were below 5% and the calibration curve contained more than three concentrations. The data and code are available in https://github.com/kruvelab/LoD_NTS.

The LoD values were estimated by using four approaches described below.

#### Cut-off Approach

The LoD was taken to be equal to the lowest concentration for which the peak was detected in at least two of the three replicates. To avoid considering a low concentration, yielding a signal indistinguishable from noise, as LoD, we furthermore evaluated that this concentration is within the dynamic range of the method.

#### Extrapolation of the S/N Approach

The second method extrapolates the S/N ratio on the lowest detected concentration within the linear range to the S/N of 3 according to eq 1:
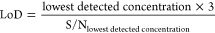
1

#### Standard Deviation-Based Approach

The third method estimates the LoD from the standard deviation (SD) of peak areas of the triplicate measurements of the lowest concentration detected as well as the slope of the calibration curve, following eq 2:
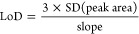
2

#### Residuals Approach

This method consists of using the slope and SD of calibration graph residuals of the three lowest concentrations following eq 3:
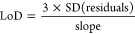
3

### Ionization Efficiency Predictions (log*IE*)

First, retention times were predicted for selected compounds using MultiConditionRT^26^ for the chromatographic conditions used in this study. This random forest model uses as an input chromatographic descriptors (pH = 2.7, acetonitrile as an organic modifier, 0.1% formic acid as the water phase, and a C_18_ reversed-phase retention mechanism), PaDEL descriptors,^27^ and log*P* values estimated using a function from the *rcdk* library. The predicted retention times were mapped to the chromatographic gradient used in this study by establishing a generalized additive model for 27 common compounds in the current study and in the original study where MultiConditionRT was introduced.^26^ In the case of known structures, the ionization efficiency was then predicted as log*IE* values using a random forest model published previously by Liigand et al.^15^ This model uses PaDEL descriptors^27^ and mobile phase descriptors (pH, viscosity, surface tension, and the presence of ammonium ions) to account for both the structure of the chemical and the mobile phase composition at the time of elution.

For the wastewater samples, log*IE* values of the unknown features were predicted using MS2Quant^28^ based on experimental retention times and molecular fingerprints obtained by SIRIUS+CSI:FingerID^29^ (version 5.8.5). These probabilistic fingerprints are predicted with support vector machines from the fragmentation trees, where the probable fragment ions are connected via neutral losses based on the information available in MS^1^ and MS^2^ spectra.

## Results and Discussion

### LoD across Approaches

Out of 221 selected compounds, 121 (54%) were detected but only 92 (42%) compounds showed a linear range with at least 3 data points and *R*^2^ > 0.90 in the standard solutions (Table S2). In the spiked wastewater samples, 55 (25%) compounds showed sufficient linearity out of 85 (38%) detected compounds (Table S3). The LoD values ranged from 1.3 × 10^–12^ to 6.7 × 10^–7^ M for the cut-off approach, 4.8 × 10^–13^ up to 1.9 × 10^–7^ M for the extrapolation of S/N, 6.1 × 10^–12^ up to 3.5 × 10^–7^ M for the standard deviation-based approach, and 7.1 × 10^–12^ up to 26.9 M for the residuals approach. The range of LoD-s values varies depending on the employed approach and its assumptions.

**Figure 1:**
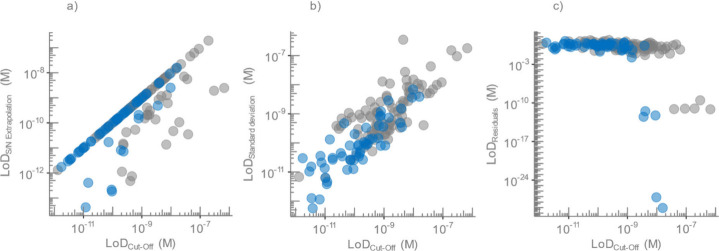
Comparison of the LoD-s determined across different approaches in standard solutions (gray dots) and spiked wastewater (blue dots). a) Cut-off approach (*x*-axis) versus extrapolation of S/N approach (*y*-axis), b) cutoff approach (*x*-axis) versus standard deviation-based approach (*y*-axis), and c) cutoff approach (*x*-axis) versus residuals-based approach (*y*-axis).

The extrapolation of S/N approach yielded lower LoD values compared to the cutoff approach (Figure 1a) for 11 compounds in the standard solutions (>10× difference). This is due to the assumption that the signal decreases linearly past the lowest experimentally studied concentration, which might be inaccurate if the lowest concentration is significantly higher than the LoD. The standard deviation-based approach yielded comparable LoD-s to the cutoff approach (Figure 1b), except for 8 compounds with higher LoD-s values (>10× difference). This might be due to the fact that the standard deviation-based approach is more sensitive to the noise (e.g., fluctuation in the ionization efficiency and ion transport). The residuals approach yielded unrealistically high LoD-s which might be due to the small number of data points in the concentration range close to the LoD and used for the calculations.^10^ Across different approaches, the observed trends aligned with the commonly accepted analytical principles (e.g., low polarity and basic compounds showing relatively low LoD in ESI+^30^ (Table S4) and early eluting compounds showing high LoD values (Table S5)).

### Association of Response Factor and LoD

The linearity of the calibration graphs was evaluated, and the response factors were calculated as the calibration graph slope for all detected compounds. The response factors ranged from 3.46 × 10^12^ to 3.21 × 10^16^ M^–1^ in standard solutions and from 2.65 × 10^14^ to 8.00 × 10^16^ M^–1^ in the spiked wastewater samples. The detected compounds showed higher response factors in spiked wastewater samples compared to the standard solutions (Figure S2); however, this increase is likely to occur due to the sensitivity shift of the instrument. Compounds lacking basic functional groups showed lower response factors (Table S6) while the highest response factors were obtained for basic compounds with p*K*_*a*_^[BH^+^]^ ≫ pH_mobile phase_ (Table S7).

**Figure 2:**
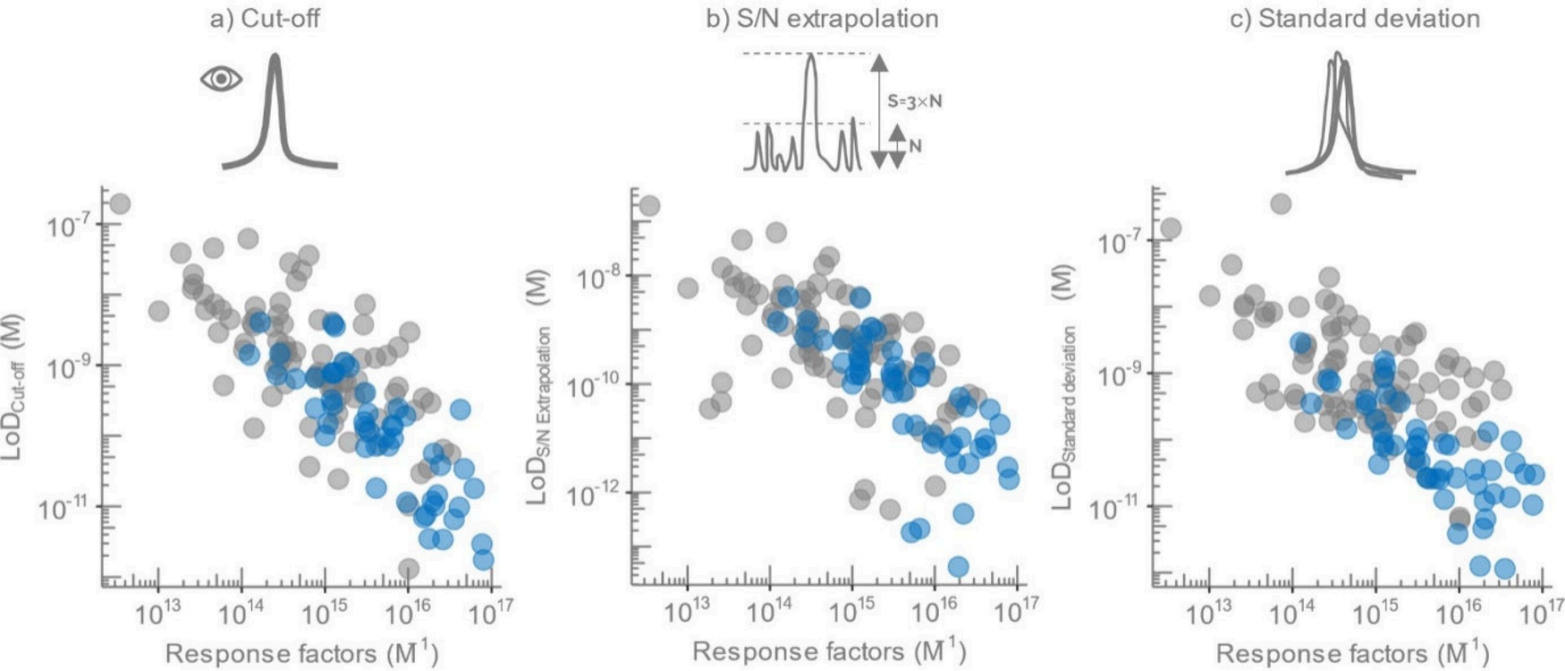
Correlation of response factors with LoD-s determined in the standard solutions (gray dots) and spiked wastewater (blue dots) using different approaches: a) cut-off approach, b) extrapolation of S/N, and c) standard deviation-based approach.

We observed that LoD values increased with decreasing response factors for all approaches (Figure 2), meaning that higher LoD values are observed for poorly ionizable chemicals and lower LoD values are observed for well-ionizable chemicals. The response factors showed correlation with the estimated LoD values for the cut-off approach, extrapolation of S/N, and standard deviation-based approaches with Spearman rho values of −0.64, −0.59, and −0.51, respectively. For all three approaches, the *p* values were below 0.05, indicating statistically significant correlation. Some outliers (e.g., penoxsulam, rimsulfuron, metsulfuron-methyl, flufenoxuron, chlorthalidone, and cefoperazone) were observed in the case of the correlation between response factors and LoD values from the extrapolation of S/N. This is likely due to the fact that this approach provides optimistically low LoD values.^10^

In the spiked wastewater samples, 85 of 221 spiked compounds were detected. The difference in detected compounds compared to the standard solutions can be due to the complexity of the matrix and its impact on ionization. After linearity evaluation and blank (nonspiked wastewater) subtraction, 30 spiked compounds remained for further evaluation (Table S3). Similarly to standard solutions, the response factors showed significant correlation with the estimated LoD values (Figure 2) based on the Spearman correlation test with *p* values <0.05 and rho values of −0.80, −0.80, and −0.74, respectively, for the cut-off approach, extrapolation of S/N, and standard deviation-based approach.

As defined above, the LoD is the smallest analyte concentration that can be reliably distinguished from the baseline. In other words, LoD is the concentration which results in a signal sufficiently higher (usually 3×) than the noise. Therefore, this concentration depends on the sensitivity of the method, which is expressed as the response factor or slope of the calibration curve, and the observed correlations are therefore expected. The correlation is imperfect as the noise also affects LoD values and the electronic and chemical noise components vary from chemical to chemical. The observed correlation of LoD values and response factors suggests that the sensitivity component is more variable across chemicals than the noise component. Moreover, the ability to predict the sensitivity for different chemicals could open up possibilities for assessing the LoD for chemicals that cannot be experimentally studied.

### Association of LoD and Ionization Efficiency

In the context of nontargeted LC/ESI/HRMS screening, the determination of response factors is limited by the availability of the analytical standards. Therefore, our group and others have suggested that the ionization efficiency (log*IE*) can be predicted either from the structure based on molecular descriptors for tentatively identified chemicals^15,31^ or from the molecular fingerprints calculated from the MS^2^ spectra for yet unidentified chemicals.^28^ We therefore hypothesize that the predicted log*IE* values may be correlated to the estimated LoD values. To test this hypothesis, log*IE* values were predicted using a random forest model presented by Liigand et al.^15^ accounting for the structure of the analyte as well as the mobile phase composition at the retention time.

**Figure 3:**
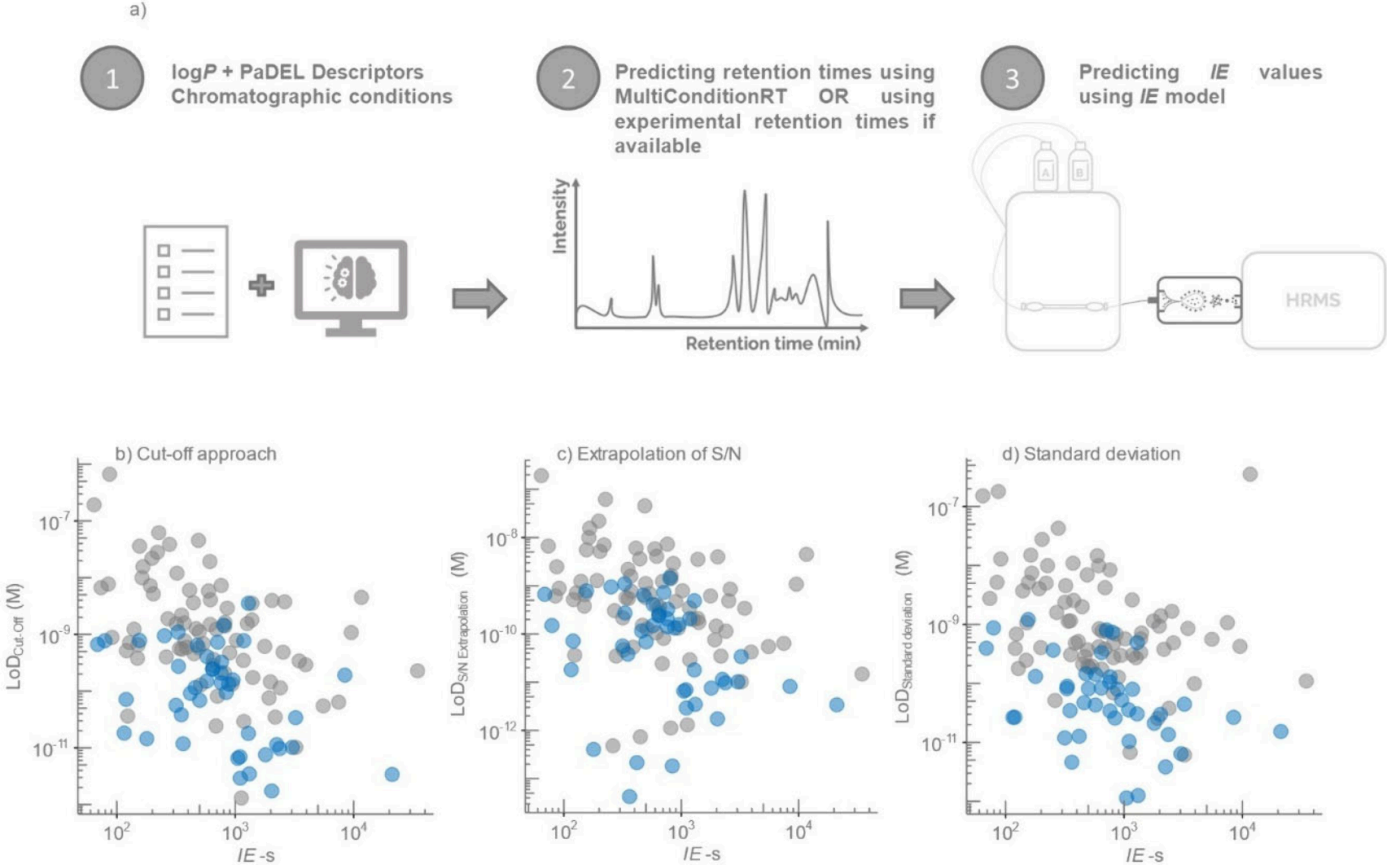
a) Schematic of the workflow to predict the ionization efficiency values. Ionization efficiency correlation with LoD-s determined in the standard solutions (gray dots) and spiked wastewater (blue dots) using four approaches: b) the correlation of *IE*-s and LoD-s from the cut-off approach, c) the correlation of *IE*-s and LoD-s from the extrapolation of S/N, and d) the correlation of *IE*-s and LoD-s from the standard deviation-based approach.

First, the log*IE* values were predicted using the experimental retention times following the workflow described in Ionization Efficiency Predictions (log IE) in the Experimental Section and Figure 3a. As the LoD values were determined for the most abundant isotopic peaks, log*IE* values were corrected to account for the main peak only. The log*IE* values, ranging from 1.78 to 4.46, showed a statistically significant correlation with the response factors based on the Spearman correlation test with a *p* value below 0.05 and rho = 0.49 (Figure S3), though the range of response factors was significantly wider (by more than 4 orders of magnitude) compared to the log*IE* range.

For the standard solutions, the predicted log*IE* values also showed a statistically significant correlation with the LoD-s determined from the cut-off approach, the extrapolation of S/N, and standard-deviation approaches (Figure 3b–d). The Spearman correlation tests yielded rho values of −0.46, −0.35, and −0.37, respectively, and all *p* values were below 0.05. This suggests that predicted ionization efficiency values can be used to roughly estimate the LoD for chemicals lacking analytical standards in LC/ESI/HRMS. A robust regression model was fitted to convert the log*IE* values to LoD values following eq 4:
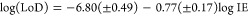
4

Another robust regression, eq 5, was fitted to describe the correlation between LoD-s and log*IE* values obtained from MS2Quant^28^ based on molecular fingerprints obtained by SIRIUS+CSI:FingerID.
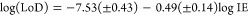
5

For applications in wastewater samples, the correlation of log*IE* and LoD values in spiked wastewater samples was investigated. The visual analysis indicated a reduced correlation while the rho values obtained from the Spearman correlation tests ranged from −0.43, −0.38, and −0.43, respectively, for cut-off, extrapolating the S/N, and standard deviation-based approaches with all *p* values <0.05 (Figure 3).

The correlation of LoD values with log*IE* values was worse than that with the response factors in the standard solutions. This might be due to multiple reasons such as the accuracy of the ionization efficiency prediction model as well as inconsistencies of the automatic integration, the small number of compounds reliably detected, and matrix effects in wastewater samples. A manual interrogation indicated that in the standard solutions acephate, irbesartan, irgarol, and clotrimazoleas as well as monocrotophos, cefoperazone, chlorpyrifos, rimsulfuron, methamidophos, nicosulfuron, tetraethylammonium, and 4-dimethylaminopyridine in spiked wastewater samples were inaccurately integrated with automatic integration; see Figures S4 and S5.

### Automatic versus Manual Integration

**Figure 4:**
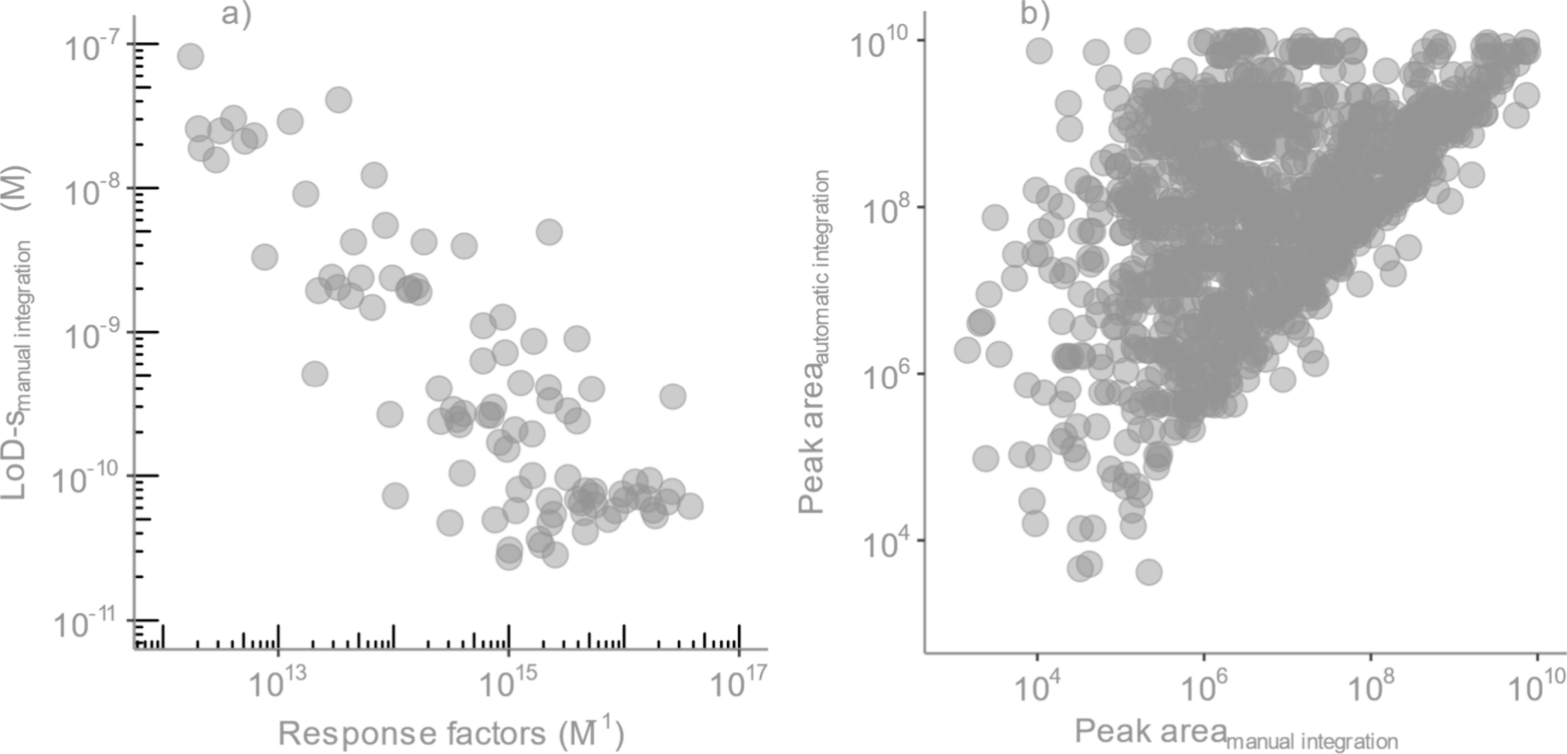
a) Correlation of calculated response factors and LoD-s from the manual integration workflow. b) Correlation of peak areas obtained from the manual integration (*x*-axis) versus automatic integration (*y*-axis).

To evaluate the impact of automatic integration on the correlation between log*IE* and LoD values, standard solutions analyzed separately under the same conditions were processed with both automated and manual workflows followed by the linearity check and the LoD estimation. Using the manual integration workflow, 104 compounds were detected and the linearity was acceptable with *R*^2^ > 0.98 for all detected compounds. The correlation between the LoD values from the cut-off approach and calculated response factors was statistically significant, with a rho value equal to −0.83 (Figure 4a). In the case of the automatic integration workflow, only 33 compounds were detected with *R*^2^ > 0.98. The correlation between LoD-s and calculated slopes yielded a rho value of −0.36 and a *p* value of 0.03 (Figure S6a). The correlation is statistically significant; however, it is reduced compared to the correlation observed for the manual integration workflow. A narrow range of concentrations was used in this case (the lowest concentration was 2.74 × 10^–11^ M); therefore, some LoD values might have been overestimated, especially for highly ionizable compounds. In addition, the manual integration yielded a wider range of response factors starting from 4.7 × 10^10^ up to 3.7 × 10^16^ M^–1^ compared to from 2.2 × 10^11^ up to 1.3 × 10^16^ M^–1^ for the automatic integration, suggesting that the compounds with low ionization efficiency and therefore small peak areas were overlooked by the automatic integration. Based on peak area comparison, the automatic integration may yield higher peak areas compared with the manual integration (Figure 4b). This highlights potential drawbacks of automatic integration on detection of low-intensity compounds as well as inconsistencies of integration, which can significantly affect the determination of LoD (Figure S6b). Nevertheless, manual integration is inaccessible for NTS with LC/HRMS where thousands of chemical features are simultaneously detected but can be appropriate in the data verification stage.

### Estimation of LoD-s for Chemical Space

**Figure 5:**
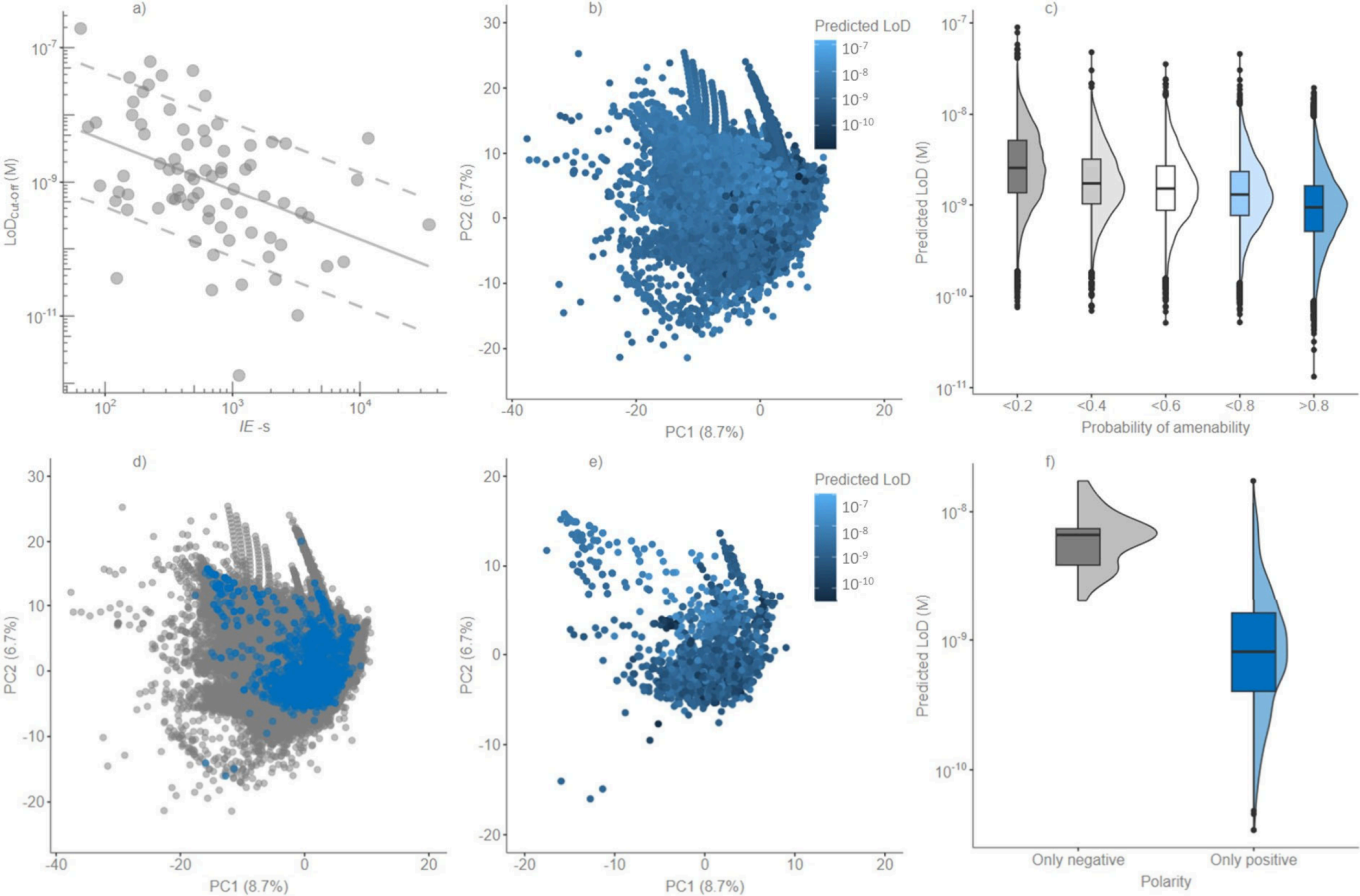
a) Robust regression model used to convert the predicted ionization efficiencies (*IE*-s) to LoD-s obtained from the cutoff approach in the standard solutions; dashed lines are used to represent the 10× error from the predicted LoD-s. b) Scores of first and second components (PC1 and PC2) of PCA fitted on the PaDEL descriptors of all compounds in Norman SusDat list (S0) colored by the estimated LoD values. c) Box and density plot of the estimated LoD-s of compounds versus probability to be amenable in ESI+ from the NORMAN SusDat list (S0). d) Scores of 2657 compounds (blue dots) projected in the same PCA fitted on S0 (gray dots). e) Scores of 2657 compounds of PC1 and PC2 colored on the basis of the predicted LoD-s. f) Box and density plot of the estimated LoD-s of the 2657 compounds grouped by the ionization mode used (positive or negative).

The NORMAN SusDat list (S0)^21^ was used to represent a chemical space of interest in environmental monitoring. It contained 70,259 compounds after preprocessing and filtering based on the *m*/*z* (100 Da as threshold) as well as the presence of carbon and nitrogen or oxygen atoms in the molecular formula. We were interested in evaluating LoD values in the ESI/HRMS positive mode for this environmentally relevant chemical space. Furthermore, as the chromatographic behavior of these chemicals is unknown, we predicted the C_18_ reversed-phase liquid chromatography retention times with the previously published MultiConditionRT^26^ model which uses descriptors of the chromatographic method (pH, organic modifier, column type) as well as compound structure described by 154 PaDEL descriptors together with log*P* values. The predicted retention times were later mapped to the chromatographic conditions used in this study, and the expected mobile phase composition at the time of elution was calculated based on this predicted retention time and the gradient used in the current study. The ionization efficiency values were predicted for all chemicals in S0 list with the model published by Liigand et al.^15^ accounting for the expected mobile phase composition at the time of elution as well as compound structure. Finally, the ionization efficiency values were converted to predicted LoD-s using the robust regression model (Figure 5a and eq 4).

The predicted LoD values ranged from 1.31 × 10^–11^ to 1.43 × 10^–7^ M for the NORMAN list (S0) (Table S8). Some of the chemicals with the highest predicted LoD values were aminomethylphosphonic acid (log *P* = −2.84, p*K*_*a*_^[BH^+^]^ = 9.94, and p*K*_*a*_^[AH]^ = −0.2), 4-methylcatehol (log *P* = 1.88 and p*K*_*a*_^[AH]^ = 9.55), *p*-nitrobenzoic acid (log *P* = 1.57 and p*K*_*a*_^[AH]^ = 3.31), and 4-methylcatechol. The high LoD-s are expected in this case, since all of these compounds are acids or contain hydroxy group and therefore are poorly ionizable in positive ESI mode. Among the chemicals with the lowest predicted LoD values were tetrahexylazanium bromide (log *P* = 4.88), tetrahexylazanium (log *P* = 4.88), tetrahexylammonium (log *P* = 4.88), and clarithromycin (log *P* = 3.24, p*K*_*a*_^[BH^+^]^ = 9, and p*K*_*a*_^[AH]^ = 12.46). These compounds contain a permanent positive charge and therefore are very ionizable in ESI.^32^

The PCA used for the selection of compounds was also used to visualize the chemical space in Figure 5b, where the scores of compounds in the first and second components are presented on the *x*- and *y*-axes and are colored by the estimated LoD values. Generally, compounds with positive scores in the first principal component (PC1) were predicted to possess lower LoD values compared to the compounds with negative scores in PC1. The analysis of the loadings plot (Figure S7) revealed that compounds with lower LoD values have high values in autocorrelation descriptors such as Geary autocorrelation descriptors (weighed by atomic mass and polarizabilities), atom type electrotopological state descriptors (such as minHBa, maxssCH2, minddssS, minsssCH, mindssC, and minaasC), and one extended topochemical atom descriptor (ETA_AlphaP). The compounds with lower LoD-s are impacted by other descriptors such as an extended topochemical atom descriptor (ETA_EtaP_F), information content descriptor IC1 (a measure of symmetry), and burden-modified eigenvalues (SpMax1_Bhs). However, general conclusions regarding the descriptors impact on detectability cannot be made due to low explained variability by PC1 (8.7%) and PC2 (6.7%). Nevertheless, PCA could be used as a visualization tool to choose chemicals and explore the chemical space with varying detectability.

Furthermore, the predicted LoD values were compared to the probability of amenability in positive ESI predicted by Alygizakis et al.^22^ Compounds with the lowest probability of amenability also yielded on average higher LoD values, while the compounds with the highest probability to be amenable yielded significantly lower LoD values (Figure 5c). The Wilcoxon rank-sum one-sided test was used to compare the mean LoD values of compounds with probability < 0.2 and > 0.8 and yielded a *p* value < 2.2 × 10^–16^; therefore, the predicted LoD approach suggested here and the probabilistic ESI amenability approach by Alygizakis et al.^22^ provide a generally agreeing indication of the detectability of chemicals in LC/ESI/HRMS. The LoD prediction approach suggested here additionally enables calibrating the detectable concentration for the instrumental method used.

Similar to the NORMAN SusDat list (S0), the LoD values were also estimated for the list of 2657 compounds that have been reported as identified at confidence level 1 or 2 in NTS studies published between 2017 and 2023 and summarized by Hulleman et al.^23^ For visualization, these 2657 compounds were also projected into the PCA of the NORMAN SusDat list (Figure 5d,e). The distribution of 2657 compounds was spread with more compounds in the center (PC1 = 0 and PC2 = 0), similar to the distribution of compounds in the NORMAN SusDat list. The estimated LoD-s ranged from 3.43 × 10^–11^ to 1.06 × 10^–7^ M with clarithromycin (log *P* = 3.24, p*K*_*a*_^[BH^+^]^ = 9, and p*K*_*a*_^[AH]^ = 12.46), azithromycin (log *P* = 2.18, p*K*_*a*_^[BH^+^]^ = 11.16, and p*K*_*a*_^[AH]^ = 12.46), amiodarone (log *P* = 7.63 and p*K*_*a*_^[BH^+^]^ = 9.08), tributylamine (log *P* = 4.16 and p*K*_*a*_^[BH^+^]^ = 11.42), and erythromycin (log *P* = 2.59, p*K*_*a*_^[BH^+^]^ = 9, and p*K*_*a*_^[AH]^ = 12.45) having the lowest LoD values. The low LoD values are expected for these compounds due to large nonpolar moieties as well as strongly basic functional groups providing a protonation site. Therefore, these compounds are expected to be retained generally in the C_18_ reversed-phase column and to be easily detectable in positive ESI. On the other hand, biphenthrin (log *P* = 6.59), adipic acid (log *P* = 0.49 and p*K*_*a*_^[AH]^ = 4.62), mannitol (log *P* = −3.73 and p*K*_*a*_^[AH]^ = 12.59), and 2,4-dinitrophenol (log *P* = 1.55 and p*K*_*a*_^[AH]^ = 4.5) had the highest LoD values, which can be explained by either the lack of a protonation site or high polarity. Hulleman et al.^23^ reported 2657 compounds detected with NTS depending on the ionization polarity. Here, compounds detected in negative ESI served only as negative control compounds that are present in the sample but have not been detected (i.e., high LoD values); therefore, comparing the LoD-s for compounds detected in positive and negative ESI was of interest. The mean predicted LoD of compounds detected only in positive ESI is significantly higher compared to the mean predicted LoD of compounds detected only in negative ESI (Figure 5f) based on the Wilcoxon test with a *p* value below 0.05.

### Estimation of LoD-s for the Prioritization of Unknown Features Detected in Wastewater Samples

In the wastewater samples, 2108 features with triggered MS^2^ spectra were extracted with MS-DIAL; however, the molecular fingerprints were calculated by SIRIUS CSI:FingerID^29^ for 1152 features only. The log*IE* values were then predicted for these 1152 features by MS2Quant^28^ and converted to LoD values with eq 5. The LoD values ranged from 3.81 × 10^–11^ to 1.54 × 10^–7^ M. The features with the 20 lowest LoD-s were extracted, and the tentative structures with the highest CSI:FingerID scores were obtained from SIRIUS+CSI:FingerID. These were ajatin; 6-heptadecyl-1,3,5-triazine-2,4-diamine (log *P* = 6.95 and p*K*_a_^[BH^+^]^ = 6.97 with multiple protonation sites); 5-octadecylpyrimidine-2,4,6-triamine (log *P* = 7.50 and p*K*_a_^[BH^+^]^ = 6.94 with multiple protonation sites); and l-leucinamide, *N*-propyl-l-isoleucyl-l-isoleucylglycyl- (log *P* = 1.66, p*K*_a_^[BH^+^]^ = 9.17, and p*K*_a_^[AH]^ = 12.2). These chemicals can be considered to be of low polarity and therefore would be retained in the C_18_ reversed-phase column as well as yield high ionization efficiency in ESI/HRMS. In addition, they have one or multiple protonation sites which are likely to contribute to high ionization efficiency in the positive ESI. No reference MS^2^ spectra were available for these compounds in MassBank;^33^ therefore, the experimental MS^2^ spectra were compared to in-silico MS^2^ spectra from MetFrag.^34^ Ajatin had two most intense peaks explained by the in-silico spectrum (*m*/*z* of 91.054 and 212.237 Da), therefore providing additional confidence for the identification on level 2. Three fragment peaks were explained for 6-heptadecyl-1,3,5-triazine-2,4-diamine; however, these peaks are of low characteristic values as they correspond to the fragments of the short alkane chain. Similar cases were observed for 5-octadecylpyrimidine-2,4,6-triamine, l-leucinamide, and *N*-propyl-l-isoleucyl-l-isoleucylglycyl-; therefore, the identification of these chemicals can be reported only at confidence level 5.

On the other hand, the features with the 20 highest LoD-s had the following tentative structures: bis(chloromethyl)carbonate; 2,4-dimethylbenzene-1,3,5-triol (p*K*_a_^[AH]^ = 10.12 and log *P* = 2.08); 1-chloro-2-methoxy-4-methyl-5-nitrobenzene (log *P* = 2.87); 5-(1,3-dithian-2-yliden)-2,2-dimethyl-1,3-dioxane-4,6-dione (log *P* = 2.46); 2,2-dinitropropanol (p*K*_a_^[AH]^ = 12.71 and log *P* = −0.23); taxicatigenin (p*K*_a_^[AH]^ = 9.46 and log *P* = 1.35); 2,4,6-trimethoxytoluene (log *P* = 2.01); vaniline (p*K*_a_^[AH]^ = 7.81 and log *P* = 1.22); 2,2-dinitropropanol (p*K*_a_^[AH]^ = 12.71 and log *P* = −0.23); and 1-phenylethyl 3-diethoxyphosphoryloxybut-2-enoate (log *P* = 3.35). These structures either are missing a basic functional group (protonation site) or are rather polar; therefore, there is a high chance that they would be detectable only at high or very high concentration levels in positive ESI or would not be retained by a C_18_ reversed-phase column. Therefore, such features can be considered unlikely and should be considered for prioritization only if additional supporting information is available. In this case, the estimated LoD can be very informative to quickly rule out false positives from SIRIUS+CSI:FingerID.

## Conclusions

A novel approach to estimating LoD values of suspected compounds for the NTS with LC/ESI/HRMS was developed to facilitate the reporting of the results and thereby improve the reproducibility. The approach estimates LoD values for suspected compounds based on the log*IE* values predicted with machine learning models from the structure or MS^2^ spectrum and accounts for the mobile phase used in LC. In this study, 78 detected compounds in the standard solutions were used to fit a robust regression with Spearman’s rho down to −0.46 between the LoD and log*IE* values. The LoD values were determined using three approaches of different complexity. The robust regression model was then used to convert the predicted log*IE*-s of suspected chemicals to LoD-s for different applications.

First, the estimated LoD-s were used to assess the general detectability of the chemicals in the NORMAN SusDat list (S0) as a chemical space of interest. In addition, the estimated LoD-s were compared to amenability probabilities in positive ESI predicted by a complementary machine learning model, and high agreement was observed. Second, the LoD-s were estimated for a dataset of 2657 compounds tentatively identified in several NTS studies between 2017 and 2023 using LC/HRMS, allowing a clear distinction of chemical space detectable in positive and negative ESI modes. Finally, the LoD-s were estimated to prioritize the unknown features detected in the wastewater samples.

## Supporting Information Available

Principal component analysis of the chemicals on the PaDEL descriptors and log P values; comparison of calculated slopes in the standard solutions and wastewater samples; comparison of the predicted ionization efficiency values and calculated response factors; examples of errors in the automatic integration for some compounds; comparison of peak areas from automatic integration and manual integration; and estimated LoD-s for the 2657 compounds detected in several NTS studies using different ionization modes (PDF)
Additional tables containing selected compounds from NORMAN SusDat list; list of detected compounds with acceptable linearity; compounds with low and high LoD-s; compounds with low response factors; and predicted LoD-s for the filtered NORMAN SusDat list (XLSX)
