# Supplementary material for: Estimating LoD-s Based on the Ionization Efficiency Values for the Reporting and Harmonization of Amenable Chemical Space in Nontargeted Screening LC/ESI/HRMS
Source: Anal Chem. 2024 Jul 3;96(28):11263–72. doi: 10.1021/acs.analchem.4c01002 (PMC11256014; doi:10.1021/acs.analchem.4c01002)
Supplement: Supplementary file 1 — ac4c01002_si_001.pdf [file ac4c01002_si_001.pdf]

Supplementary Information (SI-A)

Estimating LoD-s based on the Ionization efficiency values for reporting and harmonization in non-targeted screening LC/ESI/HRMS

Amina Souihi,[1] Anneli Kruve[1,2*]

[1]Department of Environmental and Materials Chemistry, Stockholm University, Svante Arrhenius väg 16, 106 91 Stockholm, Sweden

[2]Department of Environmental Science, Stockholm University, Svante Arrhenius väg 16, 106 91 Stockholm, Sweden

[*]Corresponding author (anneli.kruve@su.se)

Contents

Figures S2

Figure S1 Principal component analysis fitted on the PaDEL descriptors and logP values of NORMAN SusDat list (S0) S2

Figure S2 Slopes calculated in the standard solutions (x-axis) versus in the wastewater samples (y-axis) S2

Figure S3 Comparison of the slope with the predicted ionization efficiency values predicted S3

Figure S4 Errors in the automatic integration for Irbesartan S3

Figure S5 Errors in the automatic integration for Clotrimazole S3

Figure S6 a) Correlation of calculated response factors (x-axis) and LoD-s (y-axis) based on the cut-off approach using the automatic integration b) Correlation of LoD-s obtained from the manual integration (x-axis) versus automatic integration (y-axis) S4

Figure S7 Principal component analysis results S4

Figure S8 Box and density plot of the estimated LoD-s of the list 2657 compounds S5

References S6

Figures

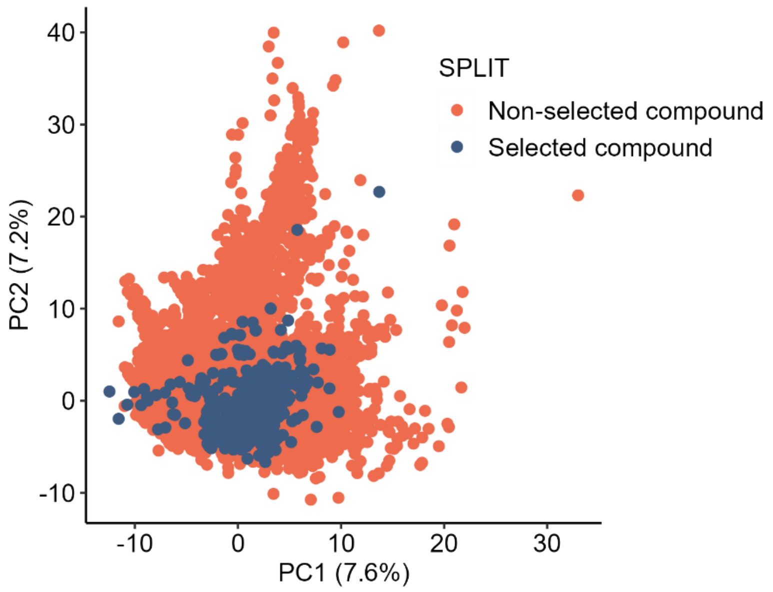

Figure S1 Principal component analysis fitted on the PaDEL descriptors and logP values of NORMAN SusDat list (S0): first component (x-axis) and second component (y-axis).

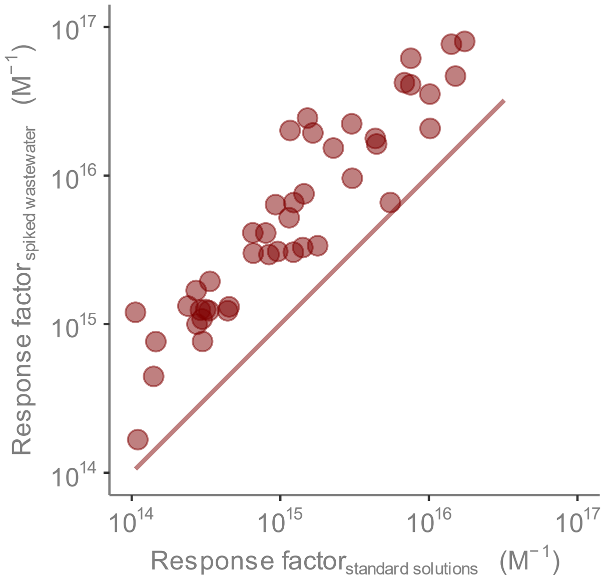

Figure S2 Slopes calculated in the standard solutions (x-axis) versus in the wastewater samples (y-axis)

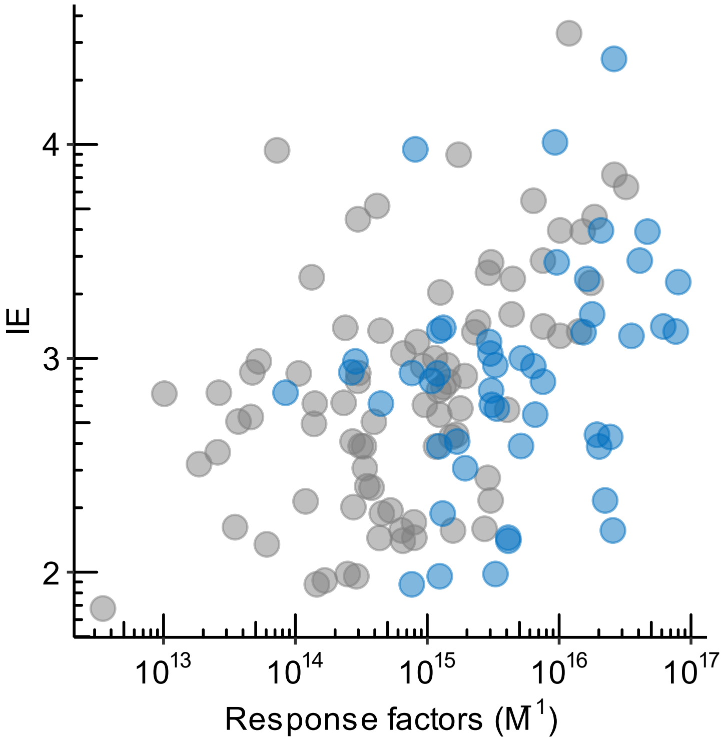

Figure S3 Comparison of the predicted ionization efficiency values and response factors calculated in the standard solutions (gray colored) and in the spiked wastewater (blue colored)

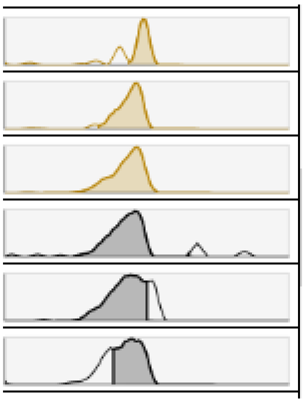

Figure S4 Errors in the automatic integration for Irbesartan

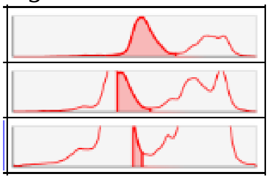

Figure S5 Errors in the automatic integration for Clotrimazole

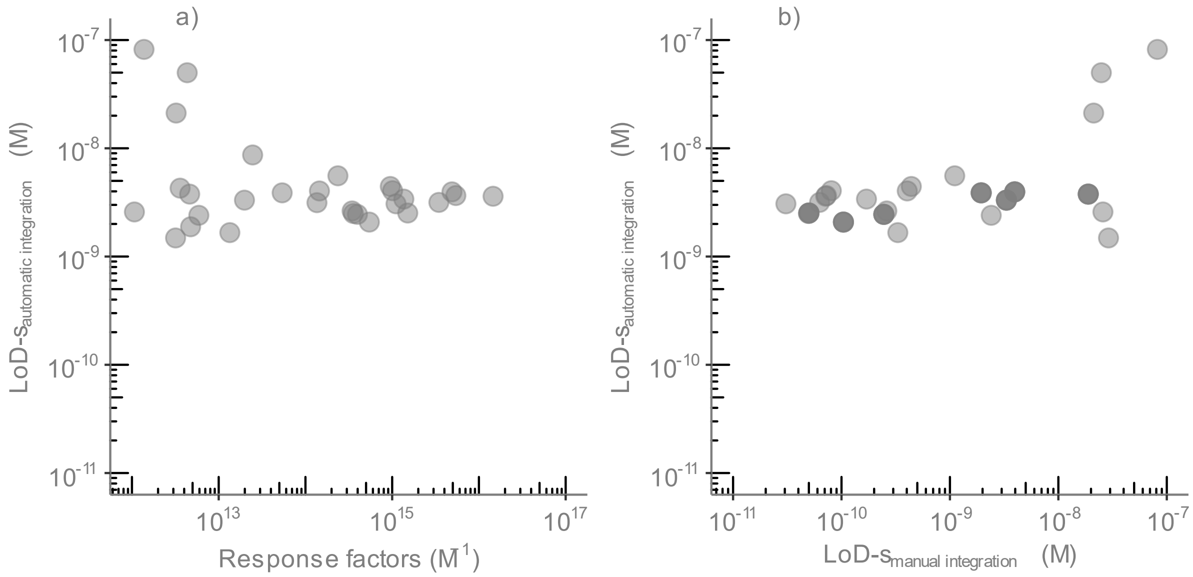

Figure S6 a) Correlation of calculated response factors (x-axis) and LoD-s (y-axis) based on the cut-off approach using the automatic integration b) Correlation of LoD-s obtained from the manual integration (x-axis) versus automatic integration (y-axis)

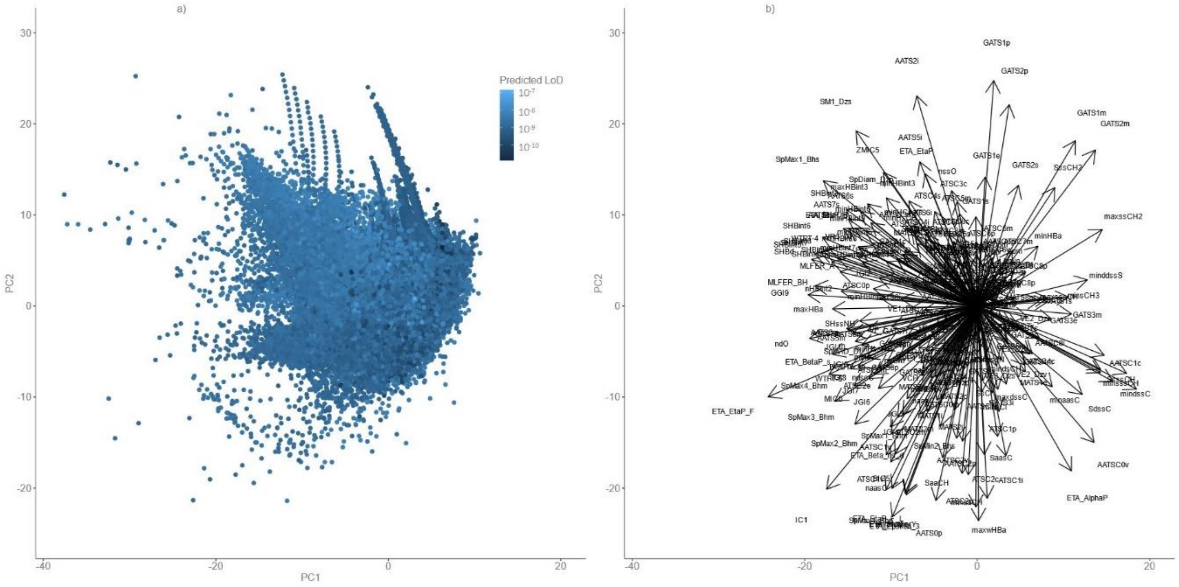

Figure S7 Principal component analysis results a) Scores of the first (x-axis) and second (y-axis) principal components of the NORMAN SusDat list[1] colored by predicted LoD-s in logarithmic scale (S0) b) Loading of the first (x-axis) and second (y-axis) components of PaDEL descriptors

.

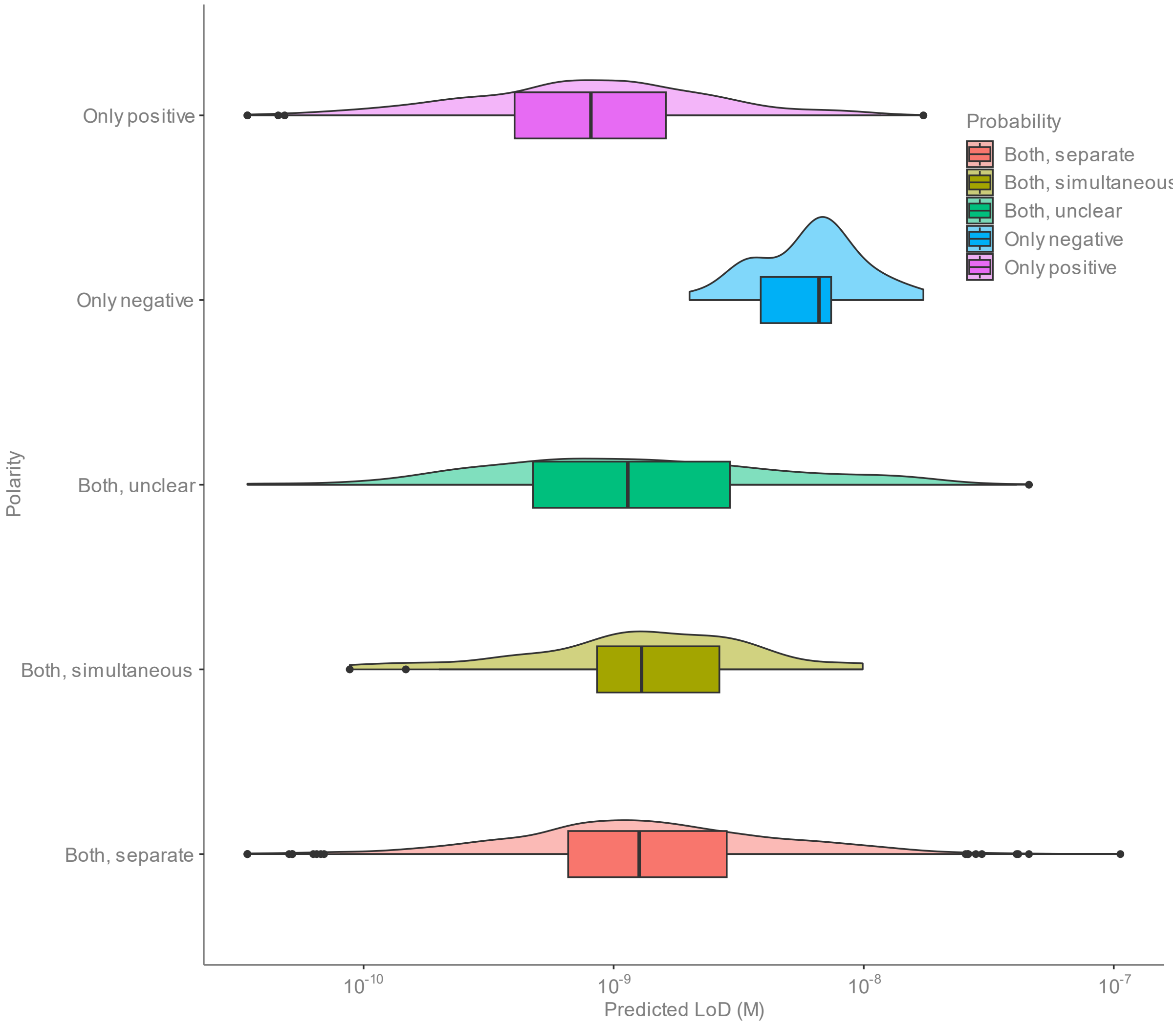

Figure S8 Box and density plot of the estimated LoD-s of the list 2657 compounds[2] grouped by the ionization mode used: positive, negative, both (unclear), both (simultaneously) and both (separately)

References

(1) NORMAN Network; Aalizadeh, R.; Alygizakis, N.; Schymanski, E.; Slobodnik, J.; Fischer, S.; Cirka, L. S0 | SUSDAT | Merged NORMAN Suspect List: SusDat, 2022. https://doi.org/10.5281/ZENODO.2664077.

(2) Hulleman, T.; Turkina, V.; O'Brien, J. W.; Chojnacka, A.; Thomas, K. V.; Samanipour, S. Critical Assessment of the Chemical Space Covered by LC–HRMS Non-Targeted Analysis. *Environ. Sci. Technol.* **2023**, *57* (38), 14101–14112. https://doi.org/10.1021/acs.est.3c03606.
